# Human resources for health planning and management in the Eastern Mediterranean region: facts, gaps and forward thinking for research and policy

**DOI:** 10.1186/1478-4491-5-9

**Published:** 2007-03-23

**Authors:** Fadi El-Jardali, Diana Jamal, Ahmad Abdallah, Kassem Kassak

**Affiliations:** 1 Health Management and Policy Department, Faculty of Health Sciences, American University of Beirut, Lebanese Republic

## Abstract

**Background:**

The early decades of the 21^st ^century are considered to be the era of human resources for health (HRH). The World Health Report (WHR) 2006 launched the Health Workforce Decade (2006–2015), with high priority given for countries to develop effective workforce policies and strategies. In many countries in the Eastern Mediterranean Region (EMR), particularly those classified as Low and Low-Middle Income Countries (LMICs), the limited knowledge about the nature, scope, composition and needs of HRH is hindering health sector reform. This highlights an urgent need to understand the current reality of HRH in several EMR countries.

The objectives of this paper are to: (1) lay out the facts on what we know about the HRH for EMR countries; (2) generate and interpret evidence on the relationship between HRH and health status indicators for LMICs and middle and high income countries (MHICs) in the context of EMR; (3) identify and analyze the information gaps (i.e. what we do not know) and (4) provide forward thinking by identifying priorities for research and policy.

**Methods:**

The variables used in the analysis were: nurse and physician density, gross national income, poverty, female literacy, health expenditure, Infant Mortality Rate (IMR), Under 5 Mortality Rate (U5MR), Maternal Mortality Rate (MMR) and Life Expectancy (LE). Univariate (charts), bivariate (Pearson correlation) and multivariate analysis (linear regression) was conducted using SPSS 14.0, besides a synthesis of HRH literature.

**Results:**

Results demonstrate the significant disparities in physician and nurse densities within the EMR, particularly between LMICs and MHICs. Besides this, significant differences exist in health status indicators within the EMR. Results of the Pearson correlation revealed that physician and nurse density, as well as female literacy in EMR countries were significantly correlated with lower mortality rates and higher life expectancy. Results of the regression analysis for both LMICs and MHICs reveal that physician density is significantly associated with all health indicators for both income groups. Nurse density was found to be significantly associated with lower MMR for the two income groups. Female literacy is notably related to lower IMR and U5MR for both income groups; and only with MMR and LE in LMICs. Health expenditure is significantly associated with lower IMR and U5MR only for LMICs. Based on results, gap analysis and the literature synthesis, information gaps and priorities were identified.

**Conclusion:**

The implication of the results discussed in this paper will help EMR countries, particularly LMICs, determine priorities to improve health outcomes and achieve health-related Millenium Development Goals.

## Background

The early decades of the 21^st ^century are considered to be the era of human resources for health (HRH). The health care sector is both labour-intensive and labour-reliant, and the delivery of quality health care services is strongly dependent on having enough well-trained health care workers to meet patient needs and expectations. The World Health Organization (WHO) estimates the current HRH workforce at 59 million and its global shortage at 4.3 million [1]. Health workers are defined as "people engaged in actions whose primary intent is to enhance health" [1]. The World Health Report (WHR), 2006, launched the Health Workforce Decade (2006–2015), with high priority given for countries to develop effective workforce strategies that include three core elements: improving recruitment, helping the existing workforce perform better, and slowing down the rate at which workers leave the health workforce. The report emphasized HRH management and planning as major strategic priorities for achieving this goal with its three core elements.

At the global level, many countries are facing critical HRH challenges including worker shortage, skill-mix imbalance, maldistribution, poor work environment, and weak knowledge base [2-4]. In several Low and Low-Middle Income countries (LMICs), the supply of health professionals is being challenged by demographic trends; an aging population; growing shortages; limited education and training capacities; poor recruitment and retention strategies including out-migration of health professionals; skill-mix imbalance; maldistribution; poor HRH planning; absence of a reliable database; poorly informed policy decisions [2,5]; and slow health system reform [5]. In Table 1, we highlight key global HRH challenges that are also relevant to LMICs.

**Table 1:** HRH challenges

**Challenges for HRH**	**Global**	**LMICs**
Health worker shortages (particularly nurses and physicians)		
Poor working conditions and remuneration		
Aging workforce		
Recruitment and retention		
Maldistribution & skill mix imbalance		
Educational reform		
Out-migration		
Health human resources planning (future needs)		
Absence of database on HRH		
Worker's health and well-being		

The HRH challenges listed in Table 1 mostly affect LMICs that suffer from poor health outcomes, such as rising death rates and decreasing life expectancies at birth [2]. This is critical in the context of the Eastern-Mediterranean Region (EMR), where the World Bank classified most (61%) of its 22 countries as Low or Low-Middle Income Countries [6]. In addition, EMR has the second lowest HRH density (per 1000 population), right after Africa, among the six administrative regions of the WHO (See Table 2) [1]. Evidence from several research studies shows that health worker density is directly correlated with population-based health indicators such as Maternal Mortality Rate (MMR), Infant Mortality Rate (IMR) and Under-5 Mortality Rate (U5MR) [7,8]. While these studies used global data to test the relationship between worker's density and health outcome indicators, none has used the most recent data to test this relationship in LMICs versus Middle and High Income countries (MHICs). While HRH density might be equally important for both LMICs and MHICs, examining the relationship for each of these two groups is critical for determining priorities for these countries to improve health outcomes and achieve the Millennium Development Goals (MDG).

**Table 2:** Density of the global health workforce across WHO administrative regions^‡^

**Region**	**Total health workforce**	
	
	**Number**	**Density (per 1,000 population)**
Africa	1 640 000	2.3
***Eastern Mediterranean***	***2 100 000***	***4.0***
South-East Asia	7 040 000	4.3
Western Pacific	10 070 000	5.8
Europe	16 630 000	18.9
Americas	21 740 000	24.8
World	59 220 000	9.3

^‡^Adapted from WHR 2006, page 5

Currently, many EMR countries are either implementing health reform plans or in the process of doing so. Evidence suggests that successful health system reform in any country depends on the provision of effective, efficient, assessable, sustainable and high quality services by a health workforce that is sufficient in number, appropriately-trained and equitably-distributed [9]. For several EMR countries, a limited understanding of HRH issues, challenges and priorities may hinder sustainable health sector reform [2,10]. Many developed countries have researched the nature and scope of HRH planning and management, particularly its problems, needs, gaps and impacts on health status. Yet for many EMR countries, almost nothing is known. This highlights an urgent need to understand the current reality of HRH in the EMR. In this paper, we make use of the most recent and available data (both global and regional) to generate and analyze evidence on HRH in the context of EMR. HRH in EMR is an underdeveloped field where evidence base has to be established. This paper will help several EMR countries determine priorities for improving population health outcomes; one of those priorities is HRH.

### Study objectives

The objectives of this paper are to:

1. lay out the facts on what we know about the HRH in EMR countries;

2. generate and interpret evidence on the relationship between HRH and health status indicators for LMICs and MHICs in the context of EMR;

3. identify and analyze the knowledge gaps;

4. provide forward thinking by identifying priorities for research and policy.

The first objective will be achieved using univariate and bivariate (Pearson correlation) analysis of the most recent regional data for the 22 EMR countries. The second objective will be realized through multivariate analysis techniques (linear regression) of the most recent global data. The remaining two objectives will be achieved by reviewing and analyzing published HRH literature in developed and developing countries. This literature includes major health reports on the EMR, published by researchers, stakeholder organizations and agencies including the WHO. To our knowledge, this study is among very few research papers that investigate HRH issues and analyze and interpret the global HRH data in the context of the EMR.

## Methods

### Study variables and sources

Our analysis comprises the following 5 independent variables:

1. Physician and nurse densities: they collectively account for the majority of healthcare providers in most countries [7];

2. Gross national income (GNI): it captures a multitude of factors that affect mortality rates such as nutrition, access to safe water, sanitation, housing, etc. [7];

3. Percentage of the population living below the poverty line of $1 per day: higher poverty rates are associated with higher mortality rates [7];

4. Female adult literacy: it is known to reflect behaviour and lifestyle which in turn influence mortality rates [7];

5. Total expenditure on health: it represents the resources spent on health, which may influence health outcomes [11].

The dependent variables are: IMR; U5MR; MMR; and Life expectancy (LE). These variables were selected since evidence shows that they can be influenced by HRH densities [1] and other socioeconomic factors. Data for both the independent and dependent variables was retrieved from the sources listed in Table 3.

**Table 3:** Sources of data used in this analysis

	**Variable**	**Source**
**Dependant variables**	IMR	World Fact Book 2005
	U5MR	World Health Report 2006
	MMR	World Health Report 2005
	LE	World Health Report 2006
**Independent variables**	Physician density	World Health Report 2006
	Nurse density	World Health Report 2006
	Female literacy	United Nations' Millennium Development Goals website
	Income	World Health Organization Statistical Information System
	Poverty	World Health Organization Statistical Information System
	Health lxpenditure	World Health Report 2006

### Methods and Data Analysis

We generated knowledge on HRH in the EMR by using data from twenty-two countries (Afghanistan, Bahrain, Cyprus, Djibouti, Egypt, Iraq, Islamic Republic of Iran, Jordan, Kuwait, Lebanon, Libyan Arab Jamahiriya, Morocco, Oman, Pakistan, Qatar, Saudi Arabia, Somalia, Sudan, Syrian Arab Republic, Tunisia, United Arab Emirates and Yemen). Only univariate and bivariate (Pearson Correlation) data analysis was performed for the EMR data due to the limited number of cases (22 countries), which does not allow the use of more advanced statistical methods such as regression analysis. To overcome this, a multivariate analysis technique was used to test the relationship between HRH and health status at the global level (all world countries) and also for LMICs versus MHICs. Countries, at a global level, were classified into these two income groups (LMICs and MHICs) based on the World Bank's (2005) income classification.

Data was regressed in three separate models: (1) at a global level, (2) for LMICs and (3) for MHICs. Poverty was dropped from all the regression models because the high percentage of missing data for this variable did not allow the models to hold (53% missing data at a global level, 38% for LMICs and 67% for MHICs). Since an initial analysis revealed a non-linear relationship between our dependent and independent variables, we estimated all regression equations within a log-linear functional form. All statistical analysis was conducted using the Statistical Package for Social Sciences (SPSS) 14.0.

## Results

**Figure 1:**
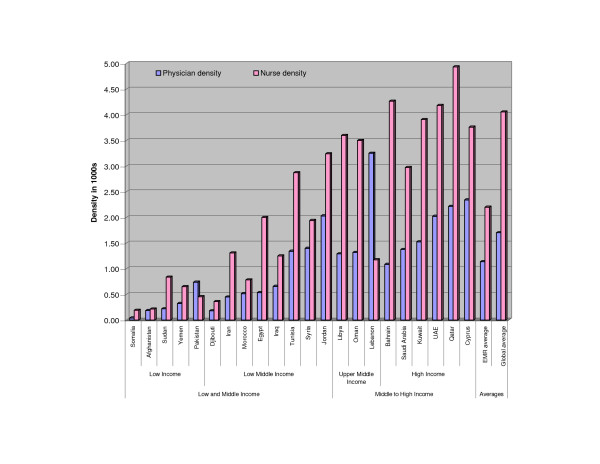
Distribution of physicians and nurses in the EMR*. *Data for nurse and physician density reflects: • 1997 estimates for Libyan Arab Jamahiriya and Somalia. • 2001 for Afghanistan, Kuwait, Lebanon, Qatar, Syrian Arab Republic and United Arab Emirates. • 2002 for Cyprus. • 2004 for Bahrain, Djibouti, Iraq, Islamic Republic of Iran, Jordan, Morocco, Oman, Pakistan, Saudi Arabia, Sudan, Tunisia, and Yemen. • 2003 for Egypt's physician density and 2004 for nurse density.

Results of the univariate data analysis indicate wide variations in terms of HRH density between the six administrative WHO regions. In fact, compared to the other regions, the EMR was found to have the second lowest HRH density (see Table 2). Even within the EMR itself, significant disparities exist concerning physician and nurse densities (see Figure 1). Of particular note is the high physician density in Lebanon compared to both the global and EMR averages. In fact, physician density in Lebanon is about twice the nurse density. Qatar is at the other end of the spectrum; its nurse density, the highest in the region, is twice its physician density.

**Figure 2:**
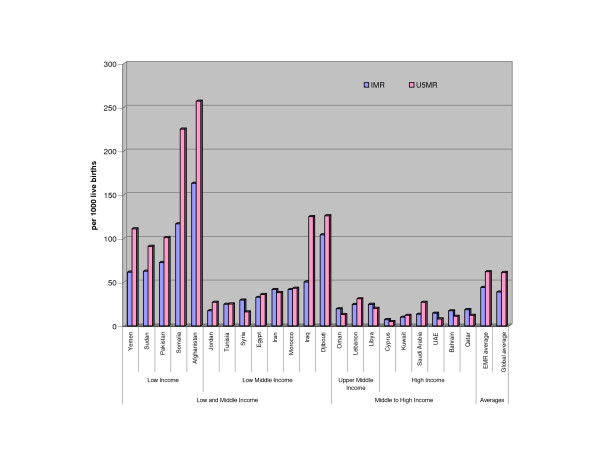
IMR (per 1000) and U5MR (per 1000) in the EMR*. *Data reflects: • 2005 estimates for IMR. • 2004 for U5MR.

Significant differences also exist in health status indicators within the (see Figure 2). Of particular interest are the cases of Somalia and Afghanistan which were observed in Figure 1 to have the lowest HRH densities in the region. The IMR in these two countries is respectively twice and thrice the regional and global averages; and their U5MR was found to be approximately four and five times the regional and global averages, respectively. This might be attributed to the recent wars in these countries and may not necessarily be a result of low HRH density. For this reason, we removed both countries from our analysis. While war conflicts exist in Iraq and Sudan as well, we did not remove them from our analysis, since their mortality rates are not as extreme as those of Afghanistan and Somalia. In fact, these rates are even lower than some other EMR countries that are not currently enduring war conflicts.

Results of the Pearson correlation revealed that physician and nurse density, and female literacy in EMR countries were significantly correlated with lower mortality rates and higher life expectancy. However, poverty, income and health expenditure were not significantly correlated with health status indicators for EMR countries (See Table 4). This latter finding runs opposite to other study findings that used global data to test such relationships [7]. This could be explained by the fact that the Pearson correlation does not allow for controlling the effect of other variables. While we were not able to perform regression analysis on the EMR data due to the limited number of cases (22 countries), we made use of the global data to test the relationship between our selected variables.

**Table 4:** Pearson correlations between HRH density and health indicators in EMR‡

	IMR	U5MR	MMR	LE
Physician density				
r	-0.695	-0.646	-0.605	0.661
Sig.	***0.001***	***0.002***	***0.005***	***0.002***
N	20	20	20	20
Nurse density				
r	-0.817	-0.794	-0.777	0.807
Sig.	***<0.000 1***	***<0.000 1***	***<0.000 1***	***<0.000 1***
N	20	20	20	20
Female literacy*				
r	-0.740	-0.746	-0.781	0.677
Sig.	***<0.000 1***	***<0.000 1***	***<0.000 1***	***<0.000 1***
N	20	20	20	20
Population living below poverty line^€^				
r	0.479	0.579	0.723	-0.511
Sig.	0.276	0.173	0.067	0.241
N	7	7	7	7
Per capita gross national income (US $)^¥^				
r	-0.323	-0.370	-0.347	0.391
Sig.	0.282	0.213	0.245	0.186
N	13	13	13	13
Total expenditure on health^₤^				
r	-0.074	-0.118	-0.058	0.051
Sig.	0.755	0.619	0.807	0.830
N	20	20	20	20

‡ Afghanistan and Somalia were found to be outliers and were therefore removed from the analysis, thus the above table is based on 20 of the EMR countries

* Data on Female literacy represents 1990 estimates for Djibouti, Iran, Lebanon, Libya, United Arab Emirates and Yemen; ad 2004 estimates for Bahrain, Cyprus, Egypt, Iraq, Jordan, Kuwait, Morocco, Oman, Pakistan, Qatar, Saudi Arabia, Sudan, Syria, and Tunisia

€ Data on population living below poverty line reflects 1997 estimates for Jordan, 1998 for Iran and Yemen, 1999 for Libya and Oman, and 2000 for Egypt and Syria

¥ Data on per capita gross national income reflects 2003 estimates

£ Data on Total Expenditure on Health reflects 2003 estimates

Regression analysis of the global data revealed that physician density was significantly associated with all health outcome indicators (see Table 5). The sign of the Beta (β) value indicates that an increase in physician density is associated with a decrease in mortality rates and an increase in LE. Increasing nurse density was only found to be significantly associated with a decrease in both MMR and LE. GNI was also significantly associated with improvement in health status indicators. Neither total health expenditure nor female literacy was significantly associated with health outcome indicators at a global level (see Table 5).

**Table 5:** Full regression analysis for predicting the influence of physician and nurse density and other socioeconomic variables on IMR, U5MR, MMR and LE at a global level

	**IMR**	**U5MR**	**MMR**	**LE**
Physician density	-0.140*	-0.248**	-0.389**	0.103**
Nurse density	0.011	0.091	-0.207*	-0.060**
Female literacy	-0.219	-0.331	-0.212	0.048
Health expenditure as % of GDP	-0.183	-0.031	-0.215	-0.030
Per capita gross national income (US$)	-0.405**	-0.515**	-0.368**	0.048**
R^2^	0.703**	0.784**	0.735**	0.709**
N	123	123	120	123

* p-value < 0.05

** p-value < 0.01

While the results from the global data analysis provide evidence that HRH density and income are important predictors of population health status in all countries, it does not provide evidence on whether such findings hold for LMICs and MHICs. Therefore, we split the global data into LMICs and MHICs and carried out the same analysis separately for each of those income groups. The importance of such examination stems from the fact that 61% of the 22 EMR countries are classified as LMICs. Thus, EMR country priorities might differ depending on its classification as LMIC or MHIC.

Results of the regression analysis for both LMICs and MHICs reveal that:

• Physician density is significantly associated with all health outcome indicators for both income groups (See Table 6); thus an increase in physician density would result in improvement in IMR, U5MR, MMR and LE.

**Table 6:** Full regression analysis for predicting the influence of physician and nurse density and other socioeconomic variables on IMR, U5MR, MMR and LE in LMICs and MHICs at a global level

	**IMR**	**U5MR**	**MMR**	**LE**
Physician density				
LMICs	-0.177**	-0.317**	-0.400**	0.101**
MHICs	-0.643**	-0.792**	-0.878**	0.186**
Nurse density				
LMICs	-0.026	0.044	-0.284*	-0.034
MHICs	-0.197	-0.238	-0.397*	-0.032
Female literacy				
LMICs	-0.573**	-0.689**	-0.491*	0.088*
MHICs	-4.277*	-5.250*	-5.051	0.344
Health expenditure as % of GDP				
LMICs	-0.433**	-0.459*	-0.348	0.023
MHICs	0.090	0.310	0.405	-0.069
R^2^				
LMICs	0.556**	0.636**	0.716**	0.615**
MHICs	0.584**	0.592**	0.485**	0.560**
N				
LMICs	93	94	93	94
MHICs	46	46	43	46

* p-value < 0.05

** p-value < 0.01

• Nurse density, on the other hand, was only found to be significantly related to lower MMR for both income groups (see Table 6).

• Female literacy, which was not significant at the global level (see Table 5), was found to be significant when data was segregated according to income level. Female literacy was associated with lower IMR and U5MR for both income groups, and with MMR and LE for LMICs.

• Health expenditure, similar to female literacy, was not significant at the global level. However, it was significantly associated with lower IMR and U5MR only at the level of LMICs.

It could thus be inferred that, in addition to physician and nurse density, female literacy and health expenditure improve health outcome indicators for LMICs. Such a finding will help EMR countries, particularly LMICs, in determining priorities to improve health outcomes and achieve health-related MDG targets.

## Discussion

Analysis of regional data revealed that LMICs in the EMR have low nurse and physician density and poor IMR and U5MR when compared to MHICs in the same region. At face value, this might imply that poor health outcome indicators for LMICs in EMR could be a product of their low HRH densities. While this justification might seem reasonable, our discussion of the results below will reveal that there are other key determinants of the poor health outcomes.

Our results pertaining to the global data analysis provide evidence that HRH density and income are important predictors of population health outcomes (IMR, U5MR, MMR, and LE) in all countries. This finding is consistent with the findings of other studies, which note that the presence of appropriate medical personnel to perform suitable medical interventions is significant for preventing the death of mothers and infants [7]. As noted earlier on our regression results, physician density is significantly associated with all health status indicators in both LMICs and MHICs. However, the lower beta (β) values for LMICs might imply that there are other critical predictors that are as important as the number of physicians in improving health outcomes in LMICs (see Table 6). Nurse density is found to be significantly associated with MMR in both LMICs and MHICs and the lower β value might be interpreted in a way similar to that of physicians.

In contrast to the findings at the global level (see Table 5), female literacy is found to be significantly associated with health outcome indicators. In LMICs, female literacy has more effect on IMR and U5MR than on MMR, as a mother's behavior has a more pronounced effect on her child's health [7]. This is demonstrated by the higher β value for IMR and U5MR than MMR and LE (see Table 6). The inverse relationship between female literacy and IMR is in accordance with the findings of Kim and Moody (1992) who found this relationship to be significant, particularly in developing countries [12].

Health expenditure is found to be significantly associated with health status indicators at the global level (see Table 5). While evidence on the association between health expenditure and health outcomes is not yet conclusive in the literature, our data analysis reveals that health expenditure is significantly associated with IMR and U5MR only in LMICs. This is of particular interest since Nixon and Ulmann (2006) suggested that a small change in health expenditure in developing countries has a bigger impact on health outcomes than a similar change in developed countries [13].

Hertz el al. (1994) documented the significant role of socioeconomic factors in improving health outcomes. Although nurse and physician density is critical, our findings, particularly those for LMICs, indicate that paying attention to socioeconomic factors such as female literacy and health expenditure is equally important for improving health outcome indicators. This finding is important for driving the performance of health systems and priority programs to achieve health-related MDG targets in EMR countries, particularly the LMICs.

### Information gaps in EMR

To reach health-related MDG and improve the performance of health systems, our analysis of the HRH facts (what we currently know from the available data) suggests that many EMR countries need to increase the number of their health workforce and adequately invest in other determinants of health, a measure that will help reduce the existing gap between the EMR and more developed regions of the world. Despite our findings confirming that the health workforce is a key factor in achieving population health goals, evidence in the literature shows that countries should not only consider the numbers, but also the management of their workforce in order to ensure adequate responses to the health system's needs. Even in those countries where the quantity of health workers is sufficient, evidence in the literature suggests that poor management of the existing health workforce will make it difficult for these workers to offer the best quality services in the most productive manner.

HRH in EMR is an underdeveloped field where it is essential to establish an evidence base. The Annual Report (2004) of WHO Eastern Mediterranean Regional Office emphasized the need for developing evidence-based guidelines for national human resources policy making, planning and management of HRH [14]. Work is in progress by the EMR regional office; its efforts are channelled to map out HRH in many countries in the region. National observatories have been established to monitor HRH development and consequently formulate regional strategies for improving HRH planning and management [15].

To better-inform HRH policies and to guide actions in terms of management and planning, essential information is needed, beyond just health worker density and health status indicators. Building on our data analysis (what we know about HRH in the EMR) and drawing on evidence on HRH from both developed and developing countries, we discuss below the third objective of this paper, which is to identify the information gaps (i.e. what we do not know) on HRH in the EMR. The information gaps are discussed in two main thematic areas: management and planning. These areas are concurrent with the 10-year plan set out by WHO (2006) for countries to improve management, recruitment and performance of HRH. Table 7 summarizes information gaps in both of these thematic areas.

**Table 7:** Information gaps in terms of management and planning for HRH

	**Information gaps**
**Management and utilization of existing HRH**	- Recruitment and retention strategies - Work conditions; training and employment characteristics, and performance - Migration and attrition - Scope of practice (underutilized or over-utilized)
**HRH planning**	- Absence of reliable HRH data (supply and needs-based) - No data numbers, gaps, losses, demographics, categories (types and skill-mix), and distribution of HRH - More comprehensive data on other categories of health workers

Work conditions can be a push or a pull factor for health workers. Heavy workloads, excessive overtime, inflexible scheduling, safety hazards, poor management and few opportunities for leadership and professional development are among the push factors that result in poor recruitment and retention of HRH, including attrition and migration. Evidence shows that good work conditions improve recruitment and retention, workers' health and well-being, quality of care and patient safety, organizational performance as well as societal outcomes. The impact of poor work conditions on recruitment and retention, worker's satisfaction, patient satisfaction, turnover rate, quality of care, patient outcomes and health systems performance is well-researched in developed countries [16,17]. Yet for countries in the EMR, almost nothing is known. In addition, no information is available on the productivity of existing health workers in this region. Literature shows that HRH shortage is more complex than a simple imbalance in supply and demand. Put simply, it is not about more supply in the short term. It is rather about effective management and better utilization of existing health workers within their legislated scope of practice [9,18]. Health care and medical knowledge are constantly evolving, which requires a clear understanding and review of existing scope of practice (i.e. the activities that health workers are educated and authorized to perform)[19]. Such information is essential in order to optimize the utilization of the existing health care workforce in the EMR, and hence control the under-and over-utilization of health workers.

In terms of HRH planning, there is limited supply-based data (i.e. numbers are only available for some categories, rather than all public health and community health workers, social workers and others). Furthermore, there is also a lack of needs-based data (i.e. the number that EMR countries need, now and in the future, to meet population health needs). Moreover, limited information is available on demographics, employment practices (full time, part time and casual), skill-mix, geographic distribution, as well as trends of migration and attrition of HRH. Errors in assembling an appropriate skill-mix can lead to clinical errors and possibly adverse patient outcomes [19]. Comprehensive data on the characteristics of health workers is therefore essential for planning, particularly at the level of conducting simulation models. These models aim at quantifying losses as well as determining how many new health workers would need to be appointed to offset the losses and estimate future needs.

### Priorities for research

While the largest component of health care costs is labour, our identification of the information gaps discussed earlier shows that little is known about this issue in the EMR countries. This represents an HRH paradox: the largest expenditure item in a health budget is the least known about in many Eastern-Mediterranean countries. For HRH policies to be effective, they should be based on and/or informed by evidence. To this end, there is an urgent need to generate research on the health workforce in the EMR.

With the current HRH data that EMR countries have, basic research questions essential for planning and management cannot be answered. In regard to effective management and utilization of existing health workers, some key research questions should be investigated and answered. Some of these questions are:

◆ How many and what type of health workers are currently available to deliver health care services in each of the EMR countries?

◆ What are the demographics of the existing HRH and how are they geographically distributed?

◆ How many health workers are required to do what, how, for whom and under what circumstances?

◆ How many new nurses, physicians and other healthcare workers are required to ensure sufficient delivery of health care services to meet the needs of the population over the next ten years (WHO's 10-year plan)?

◆ What is the right mix of health workers that can meet the health needs of the population in a given EMR country?

◆ What is known about safe-staffing, absenteeism and turnover patterns in EMR countries and how do they affect quality of care, patient outcomes and organizational performance?

◆ What were the retirement, immigration, emigration, employment and practice patterns over the last ten years or so?

◆ How many healthcare workers are expected to be lost to retirement, death and out-migration over the next ten years?

Some of the above-listed questions are well researched in developed countries; however, limited up-to-date information exists for EMR countries, especially at the level of quantity, distribution and capacities of existing HRH. Hence, health workforce research is needed in EMR countries in order to:

◆ develop a limited minimum dataset of HRH;

◆ conduct simulation models to quantify losses due to retirement, death and out-migration of HRH for the next ten years or so;

◆ determine how many new health workers would need to be appointed to offset the gap (if any); and

◆ determine how work conditions can be improved to better-recruit and retain health workers.

There is an urgent need to establish a regional research agenda, which includes feasible research questions addressing HRH issues that will likely be a priority in the EMR region two to five years from now. This period is chosen as it reflects the time required for research development and execution processes. In addition, a research synthesis agenda is required in order to address HRH priority issues over the next six to twenty-four months. This agenda recognizes the more immediate needs of policy makers, decision makers and managers for accessible summaries of existing HRH research evidence in the shorter term. This measure would assist EMR countries in developing and planning effective policies to educate, train, recruit and retain their health workforce. Priorities for research are summarized in Table 8.

**Table 8:** Priorities for research in terms of management and planning for HRH

	**Priorities for research**
**Management and utilization of existing HRH**	- Employee characteristics and productivity - Geographic distribution - Safe-staffing and workload - Absenteeism and turnover - Research on attrition and migration patterns, causes, practices and consequences
**HRH planning**	- Creating minimum database - Research on HRH numbers, gaps, losses, demographics, categories and distribution - New ways to improve data collection of stocks and flows of health workers for forecasting - Develop forecasting tools (minimum database)

### Limitations

The World Health Report 2006, titled "Working Together for Health," provides valuable data on many categories of health workers [1]. In our study, we used only physicians and nurses to represent HRH mainly because they account for the majority of care providers in most countries [7]. Another reason for not using the other categories of health workers is the large percentage of missing data, particularly for the EMR (68.2% missing data for Midwives; 63.6% for Community workers; 50% for Environmental and Public Health workers; 45.5% for Lab technicians, Health Management and support workers; and 40.9% for other categories of health workers) [1].

Data on some variables in our analysis (U5MR, MMR, LE and total expenditure on health) was initially retrieved from the WHR 2005. However, after noting considerable difference in comparison to data reported in the WHR 2006, we decided to use the more recent report to ensure reliability. The publication of the WHR 2006 offered newer, but significantly dissimilar, data than the previous report. This is due to the fact that data for most countries is estimated using regression equations and therefore, as recommended by the WHO, should be interpreted with caution. To illustrate, the WHR 2005 estimated the health expenditure in Yemen at 3.7% in 2005 and 5.5% in 2006. Lebanon's health spending as a percentage of GDP was reportedly 11.5% in 2005; it dropped down to 10.2% in 2006 [1,20]. There was also a significant difference in LE for some EMR countries. Of particular importance is the case of UAE which had an overall LE of 73 in 2005 and 77 in 2006. Although some significant year-to-year changes in the data did not lead to significant changes in our results, this variation (i.e. between WHR 2005 and WHR 2006) does reflect a need for establishing more reliable registries in EMR countries to collect and report actual data rather than estimates.

## Conclusion

The EMR has the second lowest HRH density when compared to the other WHO regions. Results demonstrate significant disparities in physician and nurse densities within the EMR, particularly between LMICs and MHICs. Besides, significant differences exist in health status indicators within the said region.

Our results strongly confirm the importance of HRH and other determinants in affecting health outcomes. An implication of our results is that investing and pouring in more money to increase the number of physicians and nurses in EMR countries – particularly the LMICs – will be less effective and, to a certain extent, wasted if not accompanied by dramatic investments in socioeconomic determinants of health. This is the case as HRH cannot be looked at in isolation from other equally important determinants of health. Investing in HRH, in addition to increasing health expenditure, expanding female education and raising national income will help countries, particularly the LMICs, to achieve health-related MDG. There are no shortcuts for achieving the health-related MDG. For LMICs, health outcome indicators will get worse, not better, if countries do not address HRH as an integral component of their health reform programs.

Achieving the MDG will not occur unless there is a right mix of health workforce with the right skills in the right place at the right time. This means that essential information beyond the mere numbers of nurses and physicians in the EMR is required. More supply of health workers in the short term may not be as effective as better-management and utilization of the existing stock of health workers in EMR countries, particularly the LMICs. For example, improving the work conditions for the existing health workforce can improve recruitment and retention, staff and patient satisfaction, quality of care and patient outcomes.

HRH issues in many EMR countries are not well researched. This paper identifies basic questions for further research. Health workforce research is needed in EMR countries in order to generate evidence to inform policy decisions, including the development of country-specific HRH policies and strategies.

## List of abbreviations in order of appearance in text

HRH: Human Resources for Health

WHO: World Health Organization

WHR: World Health Report

LMICs: Low and Low-Middle Income Countries

EMR: Eastern Mediterranean Region

MMR: Maternal Mortality Rate

IMR: Infant Mortality Rate

U5MR: Under 5 Mortality Rate

MHICs: Middle and High Income countries

GNI: Gross National Income

LE: Life Expectancy

SPSS: Statistical Package for Social Sciences

MDG: Millennium Development Goals

## Competing interests

The author(s) declare that they have no competing interests.

## Authors' contributions

FE made substantial contributions to the conception, design as well as analysis and interpretation of results. DJ made substantial contributions to acquisition, analysis of data and interpretation of results. AA made contribution to the acquisition of data and data analysis. KK made contribution to the acquisition of data in addition to revising the content of the paper. FE and DJ were involved in drafting the manuscript and revising it for intellectual content. Authors read and approved the final manuscript.
